# Macroeconomic, demographic and human developmental correlates of physical activity and sitting time among South American adults

**DOI:** 10.1186/s12966-020-01068-6

**Published:** 2020-12-14

**Authors:** André O. Werneck, Kabir P. Sadarangani, Robinson Ramírez-Vélez, Se-Sergio Baldew, Thayse N. Gomes, Gerson Ferrari, Célia L. Szwarcwald, J. Jaime Miranda, Danilo R. Silva, on behalf of the South American Physical Activity and Sedentary Behavior Network (SAPASEN) collaborators

**Affiliations:** 1 Center for Epidemiological Research in Nutrition and Health, Department of Nutrition, School of Public Health, Universidade de São Paulo (USP), Av. Dr. Arnaldo, 715 - Cerqueira César, São Paulo, SP 01246-904 Brazil; 2 Escuela de Kinesiología, Universidad Autónoma de Chile, Santiago, Chile; 3 Escuela de Kinesiología, Facultad de Salud y Odontología, Universidad Diego Portales, Santiago, Chile; 4 Department of Health Sciences, Public University of Navarra, Navarrabiomed-Biomedical Research Centre, IDISNA-Navarra’s Health Research Institute, Complejo Hospitalario de Navarra, C/irunlarrea 3, 31,008, 31008 Pamplona, Navarra Spain; 5 Department of Physical Therapy, Faculty of Medical Sciences, Anton de Kom University of Suriname, Paramaribo, Suriname; 6 Department of Physical Education, Federal University of Sergipe – UFS, São Cristóvão, Brazil; 7 Laboratorio de Ciencias de la Actividad Física, el Deporte y la Salud, Universidad de Santiago de Chile, USACH, Santiago, Chile; 8 ICICT, Fundação Oswaldo Cruz (Fiocruz), Rio de Janeiro, Brazil; 9 CRONICAS Centre of Excellence in Chronic Diseases, Universidad Peruana Cayetano Heredia, Lima, Peru; 10 School of Medicine, Universidad Peruana Cayetano Heredia, Lima, Peru

**Keywords:** Latin America, Transportation, Physical activity, Environment, Epidemiology, Global Health

## Abstract

**Background:**

Our aim was to investigate the association of macroeconomic, human development, and demographic factors with different domains of physical activity and sitting time among South American adults.

**Methods:**

We used data from nationally representative samples in Argentina (*n* = 26,932), Brazil (*n* = 52,490), Chile (*n* = 3866), Colombia (*n* = 14,208), Ecuador (*n* = 19,883), Peru (*n* = 8820), and Uruguay (*n* = 2403). Our outcomes included leisure time (≥150 min/week), transport (≥10 min/week), occupational (≥10 min/week), and total (≥150 min/week) physical activity, as well as sitting time (≥4 h/day), which were collected through self-reported questionnaires. As exposures, gross domestic product, total population, population density, and human development index indicators from the most updated national census of each country were used. Age and education were used as covariates. Multilevel logistic regressions with harmonized random effect meta-analyses were conducted, comparing highest vs. lowest (reference) tertiles.

**Results:**

Higher odds for transport physical activity were observed among the highest tertiles of total population (OR_men_: 1.41; 95% CI: 1.23–1.62), OR_women_: 1.51; 95% CI:1.32–1.73), population density (OR_men_: 1.36; 95% CI: 1.18–1.57, OR_women_: 1.49; 95% CI: 1.30–1.70), and gross domestic product (OR_men_: 1.16; 95% CI: 1.00–1.35, OR_women_: 1.39; 95% CI: 1.20–1.61). For leisure physical activity, women living in departments with higher human development index presented 18% higher odds for being active, and for total physical activity a similar estimate in both sexes was observed among those who live in more populated areas. No consistent associations were found for occupational physical activity and sitting time.

**Conclusion:**

Macroeconomic, demographic and human development indicators are associated with different domains of physical activity in the South American context, which can in turn guide policies to promote physical activity in the region.

## Introduction

Physical inactivity and sedentary behavior are associated with several chronic diseases such as cardiovascular diseases and mental disorders, as well as all-cause mortality [1–4]. Therefore, identification of the correlates of these behaviors is warranted in order to propose interventions for health promotion considering the specific characteristics of countries and regions within countries [5, 6].

 Ecological models suggest multilevel determinants of physical activity and sedentary behavior, including the role of urbanization and economic development on these behaviors [5, 7]. The association between these global-level determinants and active behaviors operate through lower-level determinants, such as policies decisions, urban planning and environment [6, 8]. For instance, more populated cities tend to be bigger and have different concerns in terms of public transportation and connectivity [9]. Also, regardless of population size, higher population density is related to higher residential density, land use mix and proximity to recreation facilities [10]. In addition, macro-level economic and human development indicators are associated to infrastructure in terms of parks, public transport, and neighborhood's aesthetics and perceived safety [5], which are important correlates of physical activity and sedentary behavior [6, 8].

Although most of the evidence of global-level determinants of active behaviors arise from high income countries, some South American countries have been through an accelerated urbanization process and are among the highest urbanized countries in the world [11], with cities presenting high population density. Moreover, South American countries tend to present inequalities within different regions inside each country [12]. Therefore, considering broader demographic and macroeconomic factors associated with physical activity and sedentary behavior should guide countries to direct interventions to those who need them the most.

Another aspect is the investigation of the various determinants that could influence people to engage in different domains of physical activity. We previously reported that higher education was positively associated with leisure-time physical activity, but inversely associated with lower transport physical activity [13]. In this sense, we need to consider country- and regional-level macroeconomic and demographic indicators among the South American countries. Previous studies, mainly focusing on developed countries, have found that higher macroeconomic, human development, and population density factors were associated with greater physical activity levels [14–18].

In South America, a region which has gone through a well-documented epidemiologic transition fueling an NCD epidemic, relatively little research has been carried out on physical activity and sitting time among adults [19]. In Europe, Cameron et al. [15] found that higher macroeconomic indicators were associated with leisure-time physical activity, but not with total physical activity. These findings indicate that macroeconomic factors can be associated with domains of physical activity, while less evidence is available regarding demographic and human development factors in South American countries.

Therefore, the present study aimed to investigate whether macroeconomic, human development, and demographic factors are associated with different domains of physical activity and sitting time among South American adults.

## Methods

### Design and sample

This is a multilevel study conducted by the South American Physical Activity and Sedentary Behavior Network (SAPASEN). SAPASEN is an initiative that aims at harmonization of national representative datasets with physical activity and sedentary behavior indicators from South America countries [20]. Our initial analysis included data from Argentina, Brazil, Chile, Ecuador, Peru, and Suriname [13, 20]. Since then, the network has expanded to include data from Colombia and Uruguay, as well as data from Chile’s most recent survey (2016–2017). Suriname was not included because we were not able to divide the health survey and census data from Suriname into geographical units (departments / regions / states). The protocols for each study were reviewed and approved locally.

We analysed data from the following national surveys: Argentina (Encuesta Nacional de Factores de Riesgo, 2013), Brazil (Pesquisa Nacional de Saúde, 2013), Chile (Encuesta Nacional de Salud, 2016–2017), Colombia (Encuesta Nacional de la Situación Nutricional, ENSIN-2010), Ecuador (Encuesta Nacional de Salud y Nutrición, 2012), Peru (Encuesta Nacional de Hogares, Módulo de Mediciones Antropométricas, 2011), and Uruguay (Encuesta Nacional de Salud, 2014). Data from each country were pooled, including participants between 18 and 64 years, except the Ecuador dataset, which included adults between 18 and 59 years. All weighted samples were calculated through complex sampling, with several levels and the common primary sample units based on the census units of each country. More detailed sampling methodology can be found in each country report [21–26] and more detailed information on the treatment and missing data for each sample are presented in the Supplementary Material A.

### Physical activity and sitting time

The International Physical Activity Questionnaire (IPAQ) [27] was used in Argentina, Ecuador, Colombia, and Peru, while the Global Physical Activity Questionnaire (GPAQ) [28] was used in Chile. Brazil used an adapted questionnaire that was based on GPAQ [22]. All questionnaires included questions regarding each physical activity domain (leisure, transportation, and occupational), except the surveys from Argentina, Colombia, and Ecuador, which did not include the occupational domain. Uruguay included questions about leisure-time domain (one question assessing the frequency of leisure-time physical activity practice - three or more, one or two, and occasionally - and one question about the time spent in the activity - 1 h or more, more than 20 min but less than 1 h, and less than 20 min). In addition, Ecuador, Brazil, Colombia, and Uruguay did not include sitting time. On the other hand, the Brazilian survey included total TV-viewing. We adopted the cut-off points of 150 min/week for leisure-time physical activity and at least 10 min/week of occupational and transport physical activity according to a previous study [13]. As there is no recommendations or commonly used cut-offs for transport and occupational domains, we used the cut-off point of at least 10 min/week based on the minimum bout of physical activity asked by IPAQ and GPAQ questionnaires, aiming to analyze people that report any activity in these domains. Despite the differences in the number of domains, which is dependent on the choice of each country, the questionnaires have slight differences especially considering the transport domains as the IPAQ asks for active transportation by bike and walking in separated questions, while the GPAQ and the Brazilian questionnaire asks for active transportation by bike and walking in the same question. The sum of the domains was used as an indicator of total physical activity. Individuals who reported more than 150 min/week of moderate to vigorous physical activity were classified as physically active, based on the WHO recommendations [29]. In addition, we adopted 4 h/day as a cut-off point for sitting time, which seems to be a critical point for increases in all-cause mortality risk [13, 30].

### Demographic, macroeconomic and human development factors

For demographic, macroeconomic and human development factors, we used data from geographic units of each country: Argentina (23 departments and Buenos Aires), Brazil (26 states and Federal District), Chile (16 regions), Colombia (32 departments and Federal District), Ecuador (24 provinces), Peru (25 regions), and Uruguay (19 departments). All the information on demographic, macroeconomic and human development factors were collected considering the most updated census of each country: Argentina: 2010; Brazil: 2010; Chile: 2017; Colombia: 2018; Ecuador: 2010 (HDI from 2017); Peru: 2017; Uruguay: 2011. The data was collected from the institutional pages of each country: Argentina: *Instituto Nacional de Estadística y Censos* (https://www.indec.gob.ar/indec/web/); Brazil: *Instituto Brasileiro de Geografia e Estatística* (https://cidades.ibge.gov.br/brasil/pa/pesquisa/37/0?ano=2010); Chile: *Instituto Nacional de Estadísticas* (https://www.ine.cl/estadisticas/sociales/censos-de-poblacion-y-vivienda); Colombia: *Departamento Administrativo Nacional de Estatística* (https://www.dane.gov.co/index.php/estadisticas-por-tema/demografia-y-poblacion/censo-nacional-de-poblacion-y-vivenda-2018); Ecuador: *Instituto Nacional de Estadística y Censos* and *Pontificia Universidad Católica del Ecuador* (https://www.ecuadorencifras.gob.ec/censo-de-poblacion-y-vivienda/); Peru: *Instituto Nacional de Estadística e Informática* (https://www.inei.gob.pe); Uruguay: *Instituto Nacional de Estadística* (https://www.ine.gub.uy/censos-2011).

We adopted total population and population density as indicators of demography as well as gross domestic product and human development index (which includes indicators of life expectancy index - life expectance at birth, education index - expected years of schooling and mean years of schooling as well as gross national income index - the gross national income) as indicators of macroeconomic and human development factors, respectively. For our approach, we adopted the internal division of each country into departments / regions / states and used the demographic, macroeconomic, and human development indicators inside each departments / regions / states (i.e. demographic, macroeconomic, and human development statistics were based on sub-regions - departments / regions / states). Subsequently, we classified total population, population density, gross domestic product, and human development index into tertiles (i.e. third as the highest tertile) [18]. The tertiles were calculated using departments / regions / states within each country (e.g. the lowest tertile of total population include participants from the department/state/region with the total population at the lowest tertile of all department/ state/ region from each country) (cut-off values are presented on Supplementary Table A). The tertiles classification was adopted to maximize the comparison between countries in the harmonization analyzes as well as to present a clearer finding of the impact of living in regions with different characteristics.

### Covariates

Chronological age (18–34, 35–49, and 50-64y), and educational status were considered as covariates in the analyses, considering that both are associated with different physical activity domains [13]. The last completed level of formal education was used to classify educational status, split into four categories: a) no formal education, b) less than secondary, c) secondary, and d) college or more.

### Statistics

Frequencies and 95% confidence intervals (95% CI) were used to describe the prevalence of each outcome as well as to compare groups [31]. Demographic, macroeconomic, and human development factors were based on geographical units (departments / regions / states). For the harmonizing process, multilevel logistic regression models, accounting for individual level and departments / regions / states level, were used to analyze the association of demographic, macroeconomic, and human development factors with domains of physical activity and sitting time. All analyses were conducted by tertiles, highest vs. lowest, with the first tertile (lowest) of each correlate as the reference group. Analyses were stratified by sex and adjusted for age group and educational level. After this, a random effect meta-analysis for multilevel logistic parameters was conducted using the command “metan” of STATA, accounting for the complex survey design in each study, calculating odds ratios (OR) and 95% confidence intervals (95% CI) for men and women, separately. Argentina and Ecuador were not included in the meta-analysis due to the lack of data on occupational physical activity. To assess the level of heterogeneity between studies, the Higgin’s *I*^2^ statistic was calculated based on country-wise estimates [32] and interpreted according previous recommendations: < 40%: might not be important, between 30 and 60%: may represent moderate heterogeneity, between 50 and 90% may represent substantial heterogeneity, between 75 and 100%: considerable heterogeneity [33]. Sampling weights were used in each study. All analyses were undertaken using STATA V.15.1 (STATA Corp, College Station, Texas, USA).

## Results

After the exclusion of participants older than 64 years and younger than 18 years as well as missing data, the final sample was composed of 128,602 adults (Argentina 26,932; Brazil 52,490; Chile 3866; Colombia 14,208; Ecuador 19,883; Peru 8820; Uruguay 2403). Characteristics of the sample according to sex are described in Table 1). The distribution of different age groups, educational status, and sex was similar across countries. The prevalence of leisure-time physical activity was slightly higher in Argentina, followed by Uruguay and Brazil (men) compared to the other countries. Colombia, Chile and Peru presented, respectively, higher levels of active transport, total physical activity, and sitting time compared to the other countries.

**Table 1:** Characteristics of the sample (*n* = 128,602)

		Country						
		Argentina	Brazil	Chile	Colombia	Ecuador	Peru	Uruguay
**Men**	Sample (n)	12,255	22,798	1400	6102	8050	3883	1075
Age group	18-34y	46.2 (44.4–47.9)	44.1 (42.9–45.2)	38.9 (34.8–43.1)	46.7 (45.1–48.3)	52.6 (50.7–54.6)	44.6 (42.5–46.7)	42.3 (38.5–46.2)
	35-49y	31.3 (29.7–32.9)	31.4 (30.4–32.5)	31.3 (27.5–35.3)	32.5 (31.1–34.1)	32.6 (30.9–34.3)	33.4 (31.5–35.3)	28.8 (25.4–32.3)
	50-64y	22.6 (21.2–24.0)	24.5 (23.5–25.5)	29.9 (26.2–33.7)	20.8 (19.5–22.1)	14.8 (13.2–16.5)	22.1 (20.4–23.8)	28.9 (25.8–32.3)
Educational status	No formal education	1.0 (0.7–1.5)	4.4 (4.0–4.9)	0.3 (0.1–0.7)	2.3 (1.9–2.8)	0.8 (0.6–1.1)	1.4 (1.1–1.8)	1.4 (0.7–2.9)
	Less than secondary	46.9 (45.1–48.6)	42.3 (41.2–43.5)	17.1 (14.4–20.1)	24.0 (22.7–25.3)	76.9 (74.6–79.0)	34.0 (32.3–35.9)	41.6 (37.9–45.5)
	Secondary education	38.7 (37.0–40.0)	39.5 (38.4–40.6)	62.1 (57.9–66.0)	57.3 (55.7–58.9)	3.5 (2.9–4.3)	44.5 (42.4–46.5)	36.1 (32.6–39.8)
	College or more	13.5 (12.4–14.7)	13.8 (13.1–14.6)	20.6 (17.2–24.4)	16.4 (15.2–17.7)	18.8 (16.8–21.0)	20.1 (18.3–21.9)	20.8 (17.9–24.1)
Physical activity	Leisure PA	34.3 (32.7–35.9)	22.1 (21.2–23.1)	23.9 (20.5–27.8)	24.0 (22.7–25.4)	23.6 (22.0–25.3)	12.8 (11.5–14.3)	29.9 (26.6–33.6)
	Transport PA	59.9 (58.0–61.8)	48.5 (47.4–49.6)	68.9 (64.9–72.6)	79.6 (78.3–80.8)	13.8 (12.5–15.3)	68.0 (65.9–69.9)	–
	Occupational PA	–	27.6 (26.6–28.7)	49.1 (44.9–53.3)	–	–	65.2 (63.1–67.3)	–
	Total PA	62.7 (61.0–64.4)	60.4 (59.3–61.5)	78.1 (74.6–81.3)	61.7 (60.1–63.2)	67.7 (65.8–69.4)	78.2 (76.2–80.1)	–
Sitting time (4 h)		59.6 (57.9–61.3)	12.4 (11.7–13.1)	39.1 (35.0–43.3)	–	–	78.0 (76.3–79.6)	–
**Women**	Sample (n)	14,677	29,692	2466	8106	11,833	4937	1328
Age group	18-34y	44.5 (42.9–46.1)	41.7 (40.7–42.7)	36.7 (33.4–40.3)	43.2 (41.8–44.5)	51.1 (49.6–52.5)	42.0 (40.2–43.8)	38.5 (35.1–41.9)
	35-49y	31.4 (30.0–32.9)	32.8 (31.9–33.8)	33.0 (29.7–36.5)	34.5 (33.2–35.8)	33.8 (32.3–35.3)	34.3 (32.6–36.0)	33.1 (29.9–36.5)
	50-64y	24.1 (22.9–25.4)	25.5 (24.6–26.4)	30.3 (27.2–33.6)	22.3 (21.2–23.5)	15.1 (13.7–16.7)	23.7 (22.2–25.4)	28.4 (25.6–31.4)
Educational status	No formal education	0.7 (0.6–1.0)	4.1 (3.7–4.4)	0.8 (0.3–2.0)	2.5 (2.2–2.9)	2.0 (1.6–2.4)	6.4 (5.7–7.2)	1.2 (0.6–2.3)
	Less than secondary	40.3 (38.7–41.8)	38.2 (37.3–39.2)	21.3 (18.6–24.1)	24.5 (23.4–25.7)	75.4 (73.7–77.1)	40.2 (38.5–42.0)	41.7 (38.3–45.2)
	Secondary education	39.8 (38.3–41.4)	41.3 (40.3–42.3)	63.6 (60.2–66.8)	57.2 (55.8–58.5)	3.8 (3.3–4.4)	34.9 (33.2–36.7)	36.9 (33.7–40.3)
	College or more	19.2 (18.0–20.4)	16.4 (15.6–17.2)	14.3 (12.1–16.9)	15.8 (14.8–16.9)	18.8 (17.3–20.4)	18.4 (17.0–20.0)	20.2 (17.6–23.0)
Physical activity	Leisure PA (≥ 150 min/wk)	24.5 (23.2–25.8)	18.7 (17.9–19.5)	13.8 (11.4–16.5)	13.0 (12.1–13.9)	7.7 (6.8–8.6)	4.7 (4.0–5.5)	21.8 (19.2–24.6)
	Transport PA (≥ 10 min/wk)	67.2 (65.4–68.8)	53.9 (52.9–54.9)	66.3 (62.9–69.6)	76.6 (75.5–77.8)	4.1 (3.5–4.8)	71.5 (69.8–73.1)	–
	Occupational PA (≥ 10 min/wk)	–	9.8 (9.2–10.4)	37.2 (33.7–40.8)	–	–	38.9 (37.2–40.7)	–
	Total PA (≥ 150 min/wk)	57.7 (56.1–59.2)	50.9 (49.9–51.9)	63.7 (60.2–67.1)	45.8 (44.5–47.2)	49.4 (47.7–51.0)	62.2 (60.4–64.0)	–
Sitting time (4 h)		57.3 (55.7–58.8)	16.9 (16.2–17.7)	31.7 (28.5–35.2)	–	–	79.5 (78.0–80.9)	–

Values are presented in percentages and 95% confidence intervals. *PA*, physical activity

**Fig. 1:**
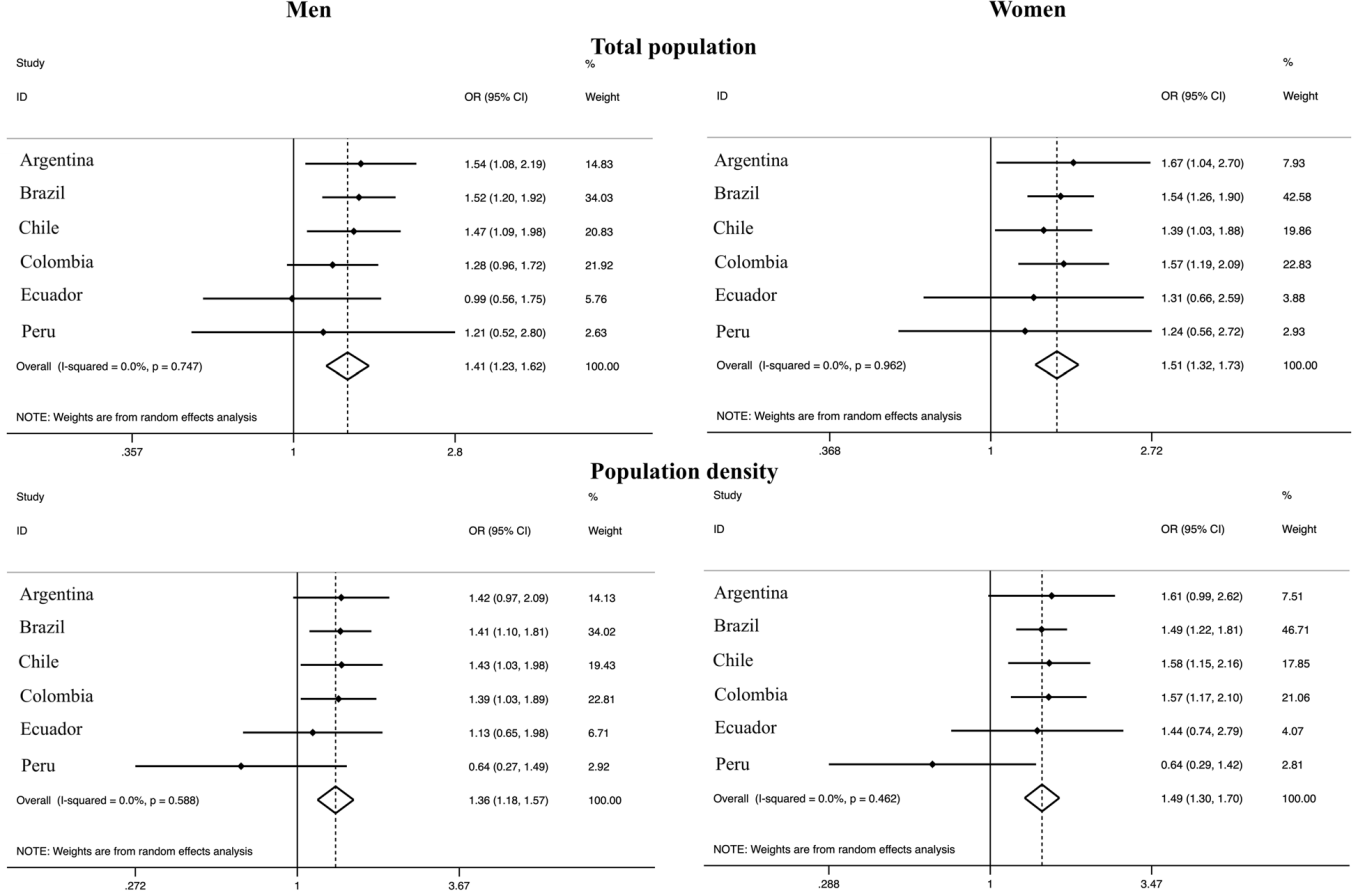
Meta-analysis of the association between different demographic factors and transport physical activity (third tertile vs. first tertile). Note. Adjusted for age and education. OR, odds ratio. CI, confidence interval. The third tertile represents the highest tertile and the first tertile represents the lowest

**Fig. 2:**
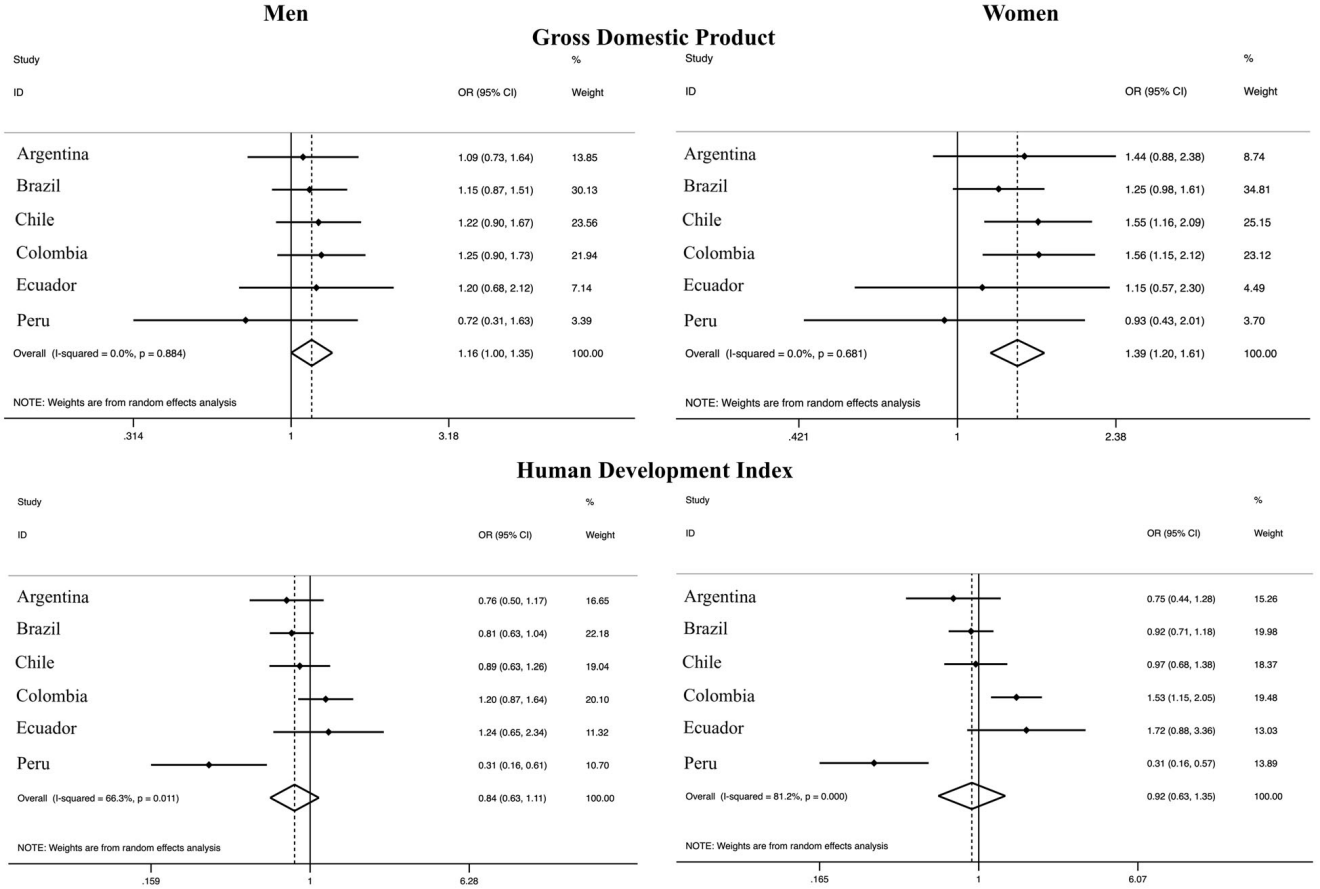
Meta-analysis of the association of different macroeconomic and human development factors with transport physical activity (third tertile vs. first tertile). Note. Adjusted for age and education. OR, odds ratio. CI, confidence interval. The third tertile represents the highest tertile and the first tertile represents the lowest

People living in more populated departments / regions / states (highest tertile) were more likely to engage in active travel [OR_men_: 1.41 (95% CI: 1.23–1.62); OR_women_: 1.51 (95% CI: 1.32–1.73)], with low variation (*I*^2^: 0% in both sexes) compared to the lowest tertile. Similarly, people living in departments / regions / states with higher population density presented higher active transport when compared with the lowest tertile [OR_men_: 1.36 (95% CI: 1.18–1.57); OR_women_: 1.49 (95% CI: 1.30–1.70)], with low variation (*I*^2^: 0% in both sexes) (Fig. 1). Higher gross domestic product was associated with higher transport physical activity [OR_women_: 1.39 (95% CI: 1.20–1.61); OR_men_: 1.16 (95% CI: 1.00–1.35)], with low variation (*I*^2^: 0% in both sexes). On the other hand, the human development index was not associated with transport physical activity (Fig. 2).

**Fig. 3:**
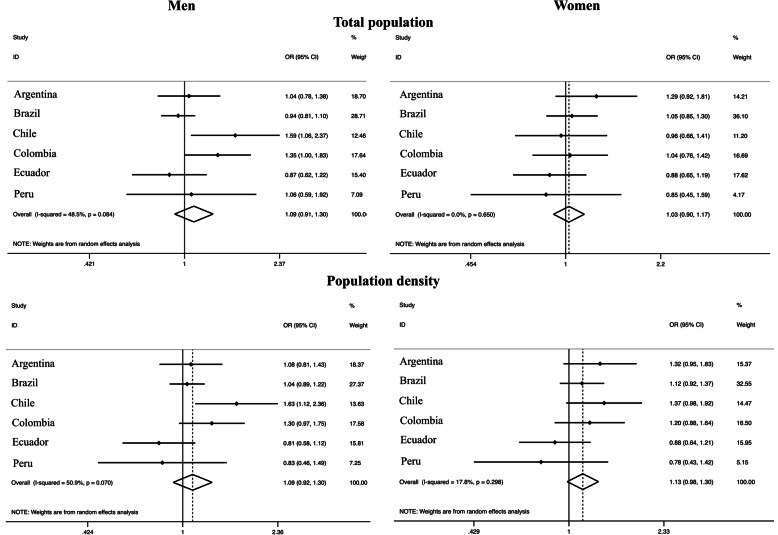
Meta-analysis of the association between different demographic factors and leisure physical activity (third tertile vs. first tertile). Note. Adjusted for age and education. OR, odds ratio. CI, confidence interval. The third tertile represents the highest tertile and the first tertile represents the lowest

**Fig. 4:**
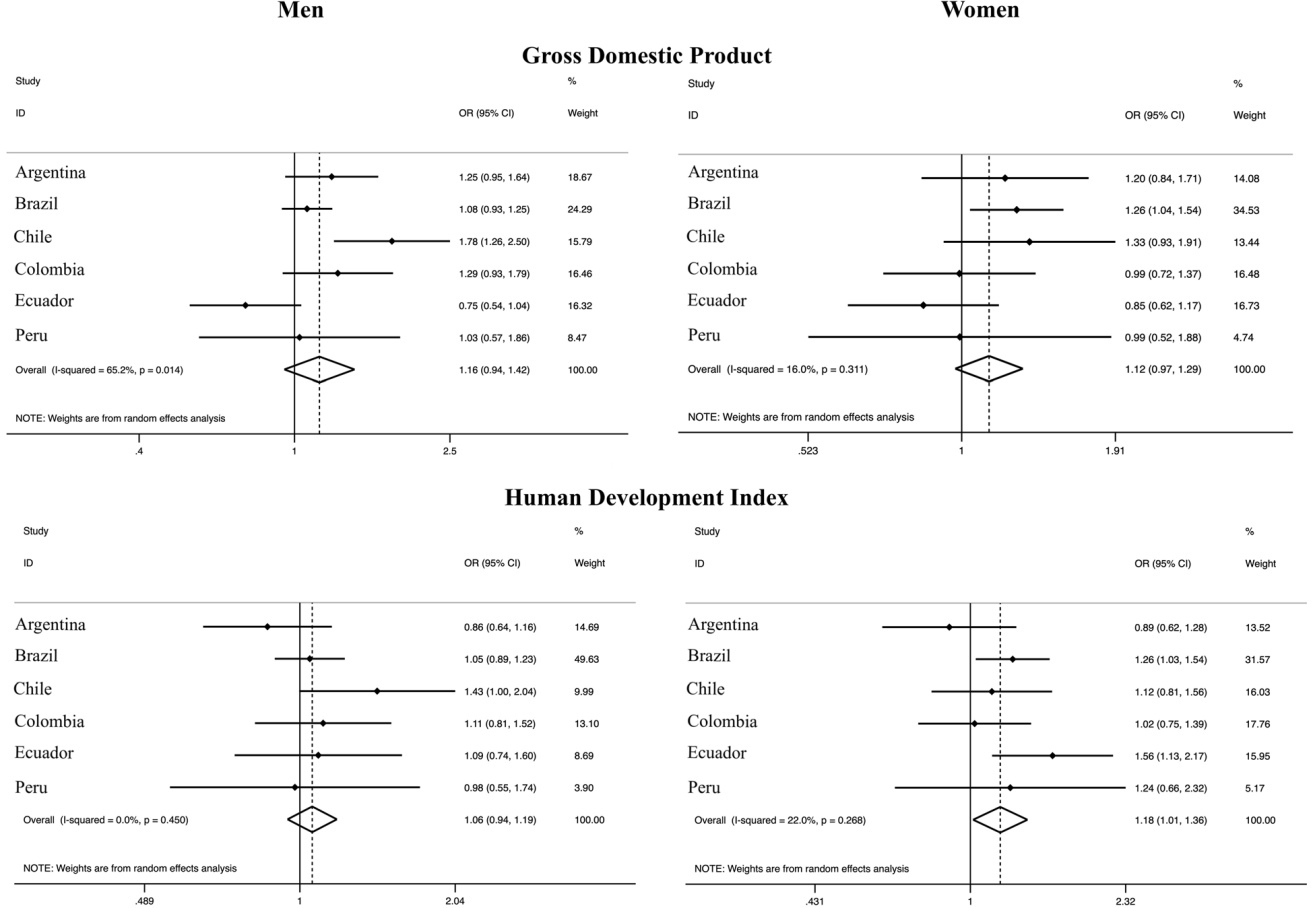
Meta-analysis of the association of different macroeconomic and human development factors with leisure physical activity (third tertile vs. first tertile). Note. Adjusted for age and education. OR, odds ratio. CI, confidence interval. The third tertile represents the highest tertile and the first tertile represents the lowest

Total population was not associated with leisure-time physical activity among both sexes and population density presented only a weak association with leisure-time physical activity among women [OR: 1.13 (95% CI: 0.98–1.30)], with low variation (*I*^2^: 17.8%) (Fig. 3). The association between the highest tertile of gross domestic product and leisure physical activity overlapped the unit in both sexes [OR_men_: 1.16 (95% CI: 0.94–1.42); OR_women_: 1.12 (95% CI: 0.97–1.29)], with a substantial heterogeneity among men (*I*^2^: 65.2%) and a trivial variation among women (*I*^2^: 16.0%). Similarly, the highest tertile of human development index was associated with higher leisure physical activity among women [OR: 1.18 (95% CI: 1.01–1.36)], with low variation across countries (*I*^2^: 22%), compared to the lowest tertile (Fig. 4).

**Fig. 5:**
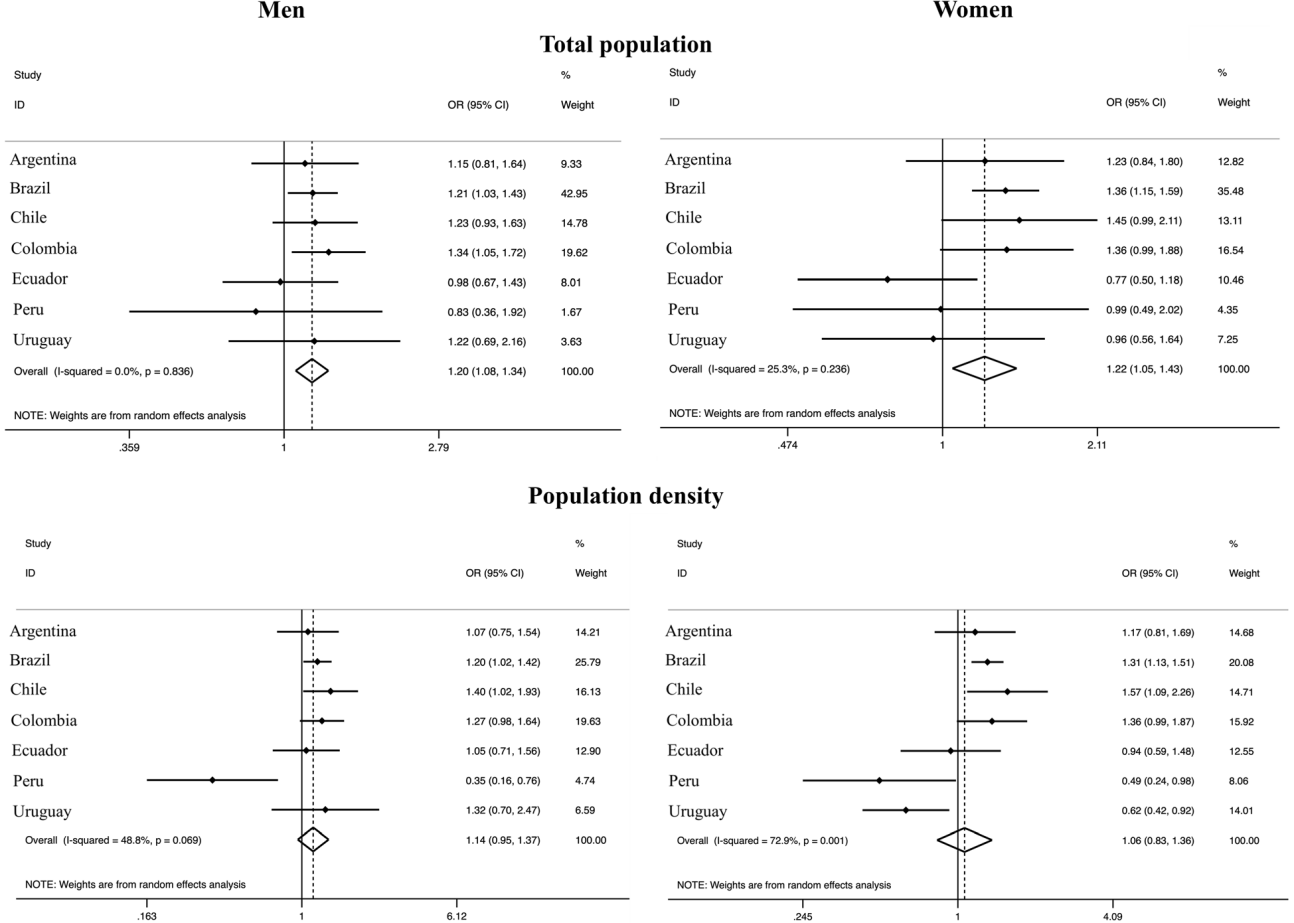
Meta-analysis of the association of different demographic factors and total physical activity (third tertile vs. first tertile). Note. Adjusted for age and education. OR, odds ratio. CI, confidence interval. The third tertile represents the highest tertile and the first tertile represents the lowest

**Fig. 6:**
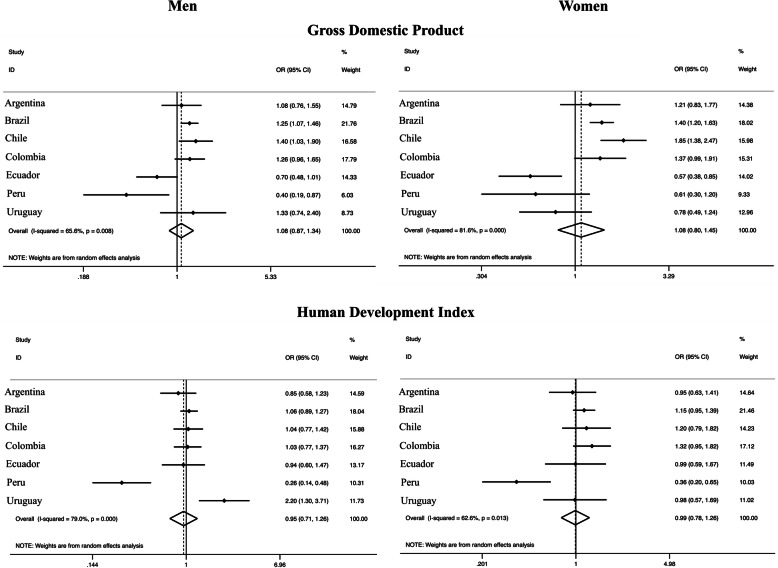
Meta-analysis of the association of different macroeconomic and human development factors with total physical activity (third tertile vs. first tertile). Note. Adjusted for age and education. OR, odds ratio. CI, confidence interval. The third tertile represents the highest tertile and the first tertile represents the lowest

Living in more populated areas was also associated with higher total physical activity in both sexes [OR_men_: 1.20 (95% CI: 1.08–1.34), I^2^:0%; OR_women_: 1.22 (95% CI: 1.05–1.43), *I*^2^: 25.3%]. Moreover, there was evidence of a weak association between population density (highest vs lowest tertile) and total physical activity among men [OR 1.14 (95% CI 0.95–1.37), *I*^2^: 48.8%] (Fig. 5). On the other hand, the gross domestic product and human development index were not associated with total physical activity (Fig. 6).

There was no evidence of an association between sociodemographic determinants and sitting time (Table 2). Regarding the association between sociodemographic determinants and occupational physical activity (Supplementary Table B), only the highest tertile of total population was associated with a higher occupational physical activity among women [OR: 1.28 (95% CI: 1.11–1.49), *I*^2^: 0%], compared to the lowest tertile. All the logistic regression comparing the middle with the lowest tertile of macroeconomic, demographic and human developmental correlates with physical activity domains and sitting time are presented on Supplementary Tables C-G.

**Table 2:** Meta-analysis of the association between different demographic, macroeconomic, and human development factors and sitting time (third tertile vs. first tertile)

	Men			Women		
	OR (95%CI)	% Weight	*I*^2^	OR (95%CI)	% Weight	*I*^2^
Total population						
Overall	0.94 (0.81–1.08)	100.00	0.0% (*p* = 0.741)	0.97 (0.83–1.14)	100.00	0.0% (*p* = 0.780)
Argentina	0.87 (0.72–1.06)	56.47		0.91 (0.71–1.16)	42.75	
Brazil	1.05 (0.81–1.35)	32.90		1.07 (0.83–1.38)	39.27	
Chile	0.95 (0.59–1.53)	9.64		0.92 (0.62–1.35)	19.88	
Peru	1.02 (0.23–4.46)	0.99		1.33 (0.29–6.18)	1.10	
Population density						
Overall	1.04 (0.85–1.29)	100.00	34.5% (*p* = 0.205)	1.10 (0.94–1.28)	100.00	0.0% (*p* = 0.413)
Argentina	1.02 (0.85–1.23)	47.16		1.12 (0.89–1.42)	44.70	
Brazil	1.20 (0.94–1.52)	38.01		1.18 (0.92–1.50)	41.38	
Chile	0.90 (0.54–1.52)	13.12		0.88 (0.57–1.36)	13.02	
Peru	0.26 (0.05–1.24)	1.71		0.39 (0.07–2.01)	0.90	
Gross Domestic Product						
Overall	1.09 (0.94–1.25)	100.00	0.0% (*p* = 0.392)	0.99 (0.84–1.17)	100.00	2.8% (*p* = 0.378)
Argentina	1.19 (0.99–1.44)	59.29		1.18 (0.91–1.53)	40.13	
Brazil	0.95 (0.73–1.22)	31.40		0.86 (0.67–1.11)	42.26	
Chile	1.00 (0.61–1.63)	8.44		0.95 (0.63–1.43)	16.39	
Peru	0.57 (0.12–2.58)	0.88		0.80 (0.17–3.70)	1.22	
Human Development Pruduct						
Overall	1.00 (0.85–1.18)	100.00	0.0% (*p* = 0.841)	0.94 (0.77–1.14)	100.00	15.2% (*p* = 0.316)
Argentina	1.08 (0.85–1.36)	48.51		1.18 (0.88–1.57)	34.78	
Brazil	0.92 (0.70–1.19)	38.56		0.82 (0.64–1.06)	41.99	
Chile	0.99 (0.62–1.60)	11.79		0.85 (0.58–1.26)	21.70	
Peru	0.87 (0.19–4.05)	1.13		0.95 (0.20–4.55)	1.53	

*OR* odds ratio, *CI* confidence interval. The third tertile represents the highest tertile and the first tertile represents the lowest

## Discussion

We aimed to investigate whether demographic, macroeconomic, and human development factors are associated with different domains of physical activity among adults from different South American countries, an important question that will allow the understanding of the burden of physical inactivity in the region. We found that a higher population and population density were associated with higher transport physical activity and total physical activity. Moreover, a higher gross domestic product was associated with higher transport physical activity and leisure physical activity, while a higher human development index was associated with higher leisure physical activity. On the other hand, we found no associations between demographic, macroeconomic, and human development determinants and sitting time.

Previous studies investigated the association of demographic, macroeconomic, and human development determinants on physical activity and sitting time [14–18]. However, these studies investigated country-level macroeconomic, demographic, and human development determinants, with the inherent limitations that countrywide averages carry. Given the major within-country disparities in South America [12], this study advances exploration of an within-country variation across a number of macroeconomic, demographic, and human development determinants.

South America passed through an accelerated urbanization process, which resulted in specific distribution of cities, with a high population density pattern and infrastructure deficiency for physical activity [34]. These processes can bring broader correlates to the understanding of different health behaviors such as physical activity and sitting time [6, 34]. We confirmed the consistent association of total population and population density with higher transport physical activity in both sexes [35]. In this sense, different components of higher urbanization could be associated with higher transport physical activity, such as higher connectivity, a more densely connected environment, and mixed-use land [6, 36]. Moreover, cities with a higher population and population density tend to present more developed public transport systems, which could also be associated with higher transport physical activity [37, 38].

The association between macroeconomic and human development factors and leisure-time physical activity highlights the access to environments for physical activity practice and its impact on this domain. This finding is in line with a previous study investigating European countries [15]. Regions with a higher gross domestic product as well as a higher human development index can invest in the prioritization and maintenance of parks and green areas [39], which are associated with higher levels of physical activity, especially leisure time-related activities [36, 40, 41]. Moreover, characteristics such as better quality streets, community safety, and even the design of the cities can also encourage the practice of physical activity [6, 42].

There were no consistent associations of different demographic, macroeconomic, and human development factors with sitting time and occupational physical activity. The lack of association regarding occupational physical activity could indicate that individual-level correlates, such as socioeconomic condition could better explain the variation in this domain of physical activity. Likewise, with regards to sitting time, previous studies found that environmental macro-determinants of sitting time are less consistent than individual determinants [43]. Therefore, policies to enhance physical activity and mobility behaviors need to recognize and tailor the specific challenges of both promoting physical activity and actively reducing sitting time.

The present study advances the regional level approach of investigating areas with a higher risk for physical inactivity. When analyzing total physical activity, less populated and dense areas from South America should emphasize the formulation of intervention strategies. On the other hand, the emphasis on specific domains of physical activity should be given consideration; particularly the inequality regarding the total population and population density on transport physical activity, which was also consistently lower in low populated areas. Our findings show that, leisure physical activity was dependent on macroeconomic and human development factors, such as gross domestic product and human development index, in which areas with a lower gross domestic product and human development index presented lower rates of leisure physical activity. Therefore, the stimulation of leisure physical activity should be heavily promoted on regions with lower socioeconomic indicators. Assuming these ecological correlations are causal, this geographical connection suggests that certain strategies (e.g., global physical activity guidelines [44]) promoting the reduction in sitting time and increase in moderate to vigorous physical activity might be better achieved through population-wide approaches rather than focusing on individual recommendations alone.

Some limitations should be considered in the interpretation of our findings. The study design does not allow inference about causality and these findings should be interpreted considering this. Different surveys assessed physical activity and sitting time through the use of different self-report questionnaires, and these instruments could be prone to potential recall bias. Moreover, there was a 6 year range from different surveys (2010–2016), which could also introduce additional biases, however a previous study did not find a trend in physical inactivity within this period [45]. Although we used data from the most recent census and representative samples that measured physical activity, the year of the representative dataset of physical activity and census was not the same. Despite this, our study differs from previous studies, as our final indicators for demographic, macroeconomic, and human development factors were based on sub-regions (departments / regions / states), which should be considered a strength of this study. Also, there are potential confounders that we were not able to adjust in the analysis due to the lack of availability in different surveys, such as income. Finally, the analysis of total physical activity should be inferred with caution, because different countries adopted different domains in their questionnaire. The present analysis represents approximately 89% of the South American population, a considerable increase when compared with the previous estimates from SAPASEN – (from 76%), with the differences derived from the inclusion of data from Colombia and Uruguay [13].

To the best of our knowledge, this is the first multi-country study in South America to identify distribution of physical activity and sitting time according regional macro-level determinants. These findings should reach regional health sectors and policy makers, informing physical activity plans according to the regional characteristics. For example, less populated regions should invest in strategies to improve active transportation, such as multimodal transport models. For regions with higher population and population density, where people are more active in transportation, investments on better sidewalks and bike paths should be prioritized in order to improve infrastructure and perceived safety during transportation. Also, infrastructure for leisure-time physical activity as the creation of parks should be stimulated in areas with lower HDI and GBD aiming to increase opportunities to leisure-time activities.

We conclude that, in the South American setting higher population and population density are associated with higher transport physical activity, while higher gross domestic product and human development index are associated with higher leisure-time physical activity. There were no associations observed with sitting time.

## Supplementary Information

**Additional file 1.**

## Data Availability

All datasets are available in each governmental website.
