# Supplementary material for: Macroeconomic, demographic and human developmental correlates of physical activity and sitting time among South American adults
Source: Int J Behav Nutr Phys Act. 2020 Dec 14;17:163. doi: 10.1186/s12966-020-01068-6 (PMC7737374; doi:10.1186/s12966-020-01068-6)
Supplement: Supplementary file 1 — Additional file 1. [file 12966_2020_1068_MOESM1_ESM.docx]

**Supplementary Material A**

Sampling and data cleaning of the used surveys:

**Argentina:** Encuesta Nacional de Factores de Riesgo, 2013

The *Encuesta Nacional de Factores de Riesgo* sampling was through different stages, stratified by conglomerates. The statistical unit was the living house; the relevance unit, the home; and the analysis unit, the individual. The sample size selected was 46 555 individuals, of which any number could be surveyed at 32,365 (response rate: 70.7%). From the 32,365 included in the sample, 5,376 participants were older than 64 years or younger than 18 years. Also, considering missing data, the final sample was composed of 26,932 adults. More details can be found in the official report [1].

**Brazil:** Pesquisa Nacional de Saúde 2013

The *Pesquisa Nacional de Saúde* sampling was through different stages. Firstly, census tracts were randomly selected, next, households were randomly selected; and finally, in the households, one adult was randomly selected. The minimum sample size per federal unit (n = 27) was 1800 households. From the planned 69,954 households, interviews were conducted in 64,348 households. Due to missing data, 60,202 participants were included in the final sample (response rate: 86%). Due to our analysis, 7,712 participants were also excluded because they were outside the age range (Final sample: 52,490). More details can be found in the official report [2].

**Chile:** Encuesta Nacional de Salud, 2016-2017

The *Encuesta Nacional de Salud*, 2016-2017 sampling was through different stages, considering the different strata (regions), households and the selected participant. The initial plan was to include 6,027 participants, considering the sample size calculations. For this, it was adopted an oversample (10,124 households). The final sample size was composed of 6,233 adults. From the initial sample, 1,755 were excluded because were older than 64y or younger than 18y. In addition, due to missing data, the final sample was composed of 3,866 adults. More details can be found in the official report [3].

**Colombia:** Encuesta Nacional de la Situación Nutricional, ENSIN-2010

The *Encuesta Nacional de la Situación Nutricional*, 2010 sampling was through different stages. Firstly the cities from all departments of Colombia were grouped in strata, considering similar sociodemographic characteristics. After this, cities were ramdomly selected within each strata, considering the keeping the proportional probability of the stratum size. In each strata, 10 households were randomly selected and all members were invited to take part in the study. From the final sample, a representative sub-sample was selected to report physical activity levels. The initial estimated sample size was 15,000, but the final sample was composed of 14,465 adults between 18 and 64 years, residents in urban areas. Considering missing data, the included sample was 14,208. More details can be found in the official report [4].

**Ecuador:** Encuesta Nacional de Salud y Nutrición, 2012

The *Encuesta Nacional de Salud y Nutrición*, 2012 sampling was through different stages. The sampling process considered the 24 provinces as strata and then, the primary sampling unit was the census sector, the secondary was housing and the third are target population groups from 0 months to 59 years**.** The whole sample was composed of 92,502 participants, with valid data in at least one variable. However, the module of physical activity was applied only in adults between 18 and 59 years, living in urban areas. Therefore, the final sample was composed of 19,883 adults. More details can be found in the official report [5].

**Peru:** Encuesta Nacional de Hogares, Módulo de Mediciones Antropométricas, 2011

The *Encuesta Nacional de Hogares*, Módulo de Mediciones Antropométricas used a probabilistic, stratified and multistage sampling in each department of Peru. The sampling was based on the sampling from the *Encuesta Nacional de Hogares* (National Household Survey). 5,792 homes were visited (3,384 in urban areas and 2408 in rural areas) and 10,717 participants were evaluated (between 15 and 69 years). Considering the exclusion due to the focused age range (18-64 years) and missing data, the final sample was composed of 8,820 adults. More details can be found in the official report [6].

**Uruguay:** Encuesta Nacional de Salud, 2014

The sampling process of the *Encuesta Nacional de Salud* was composed of multiple stages, aiming to include representative sample of inhabitants from cities of at least 5,000 residents. Cities were selected (proportional to the population size), considering the departments as strata. Small cities (between 5,000 and 10,000 inhabitants) were sampled separetely due to the lower probability of selection. Census sectors were ramdomly selected in each selected city and the residents of each house were invited to take part in the study. The final sample was composed of 3,543 participants (with 15 years or older). Considering the age range of the present study (18-64 years), the included sample was composed of 2,403 adults. More details can be found in the official report [7].

**References**

1. Ministerio de Salud. *Tercera Encuesta Nacional de Factores de Riesgo Para Enfermedades No Transmisibles*.; 2013.

2. Instituto Brasileiro de Geografia e Estatística - IBGE. *Pesquisa Nacional de Saúde 2013*.; 2014.

3. Departamento de Epidemiología División de Planificación Sanitaria Subsecretaría de Salud Pública. *ENCUESTA NACIONAL DE SALUD 2016-2017*.; 2017.

4. Instituto Colombiano de Bienestar Familiar. *Encuesta Nacional de La Situación Nutricional En Colombia 2010 ENSIN*.; 2010.

5. Ministerio de Salud Pública. *Encuesta Nacional de Salud y Nutrición ENSANUT-ECU 2012*.; 2012.

6. Instituto Nacional de Estadística e Informática (INEI). *Encuesta Nacional de Hogares 2011 - Condiciones de Vida y Pobreza*.; 2011.

7. Ministerio de Salud. *Encuesta Nacional de Salud Uruguay*.; 2016.

**Supplementary table A**. Cut-off points adopted for each indicator in each country, considering the tertile distribution of state/department/region inside each country.

|  | **Country** | | | | | | |
| --- | --- | --- | --- | --- | --- | --- | --- |
|  | **Argentina** | **Brazil** | **Chile** | **Colombia** | **Ecuador** | **Peru** | **Uruguay** |
| **Total population** |  |  |  |  |  |  |  |
| *Middle tertile, n* | 551 266 | 3 119 015 | 384 837 | 509 640 | 183 641 | 496 459 | 82 595 |
| *Highest tertile, n* | 1 214 441 | 7 603 239 | 957 224 | 1 263 788 | 491 168 | 1 197 260 | 123 203 |
| **Population density** |  |  |  |  |  |  |  |
| *Middle tertile, hab/km²* | 5.9 | 12.4 | 13.4 | 39.6 | 40.7 | 19.5 | 6.4 |
| *Highest tertile, hab/km²* | 12.7 | 65.3 | 34.5 | 73.5 | 86.6 | 49.7 | 21.7 |
| **Gross Domestic Product** |  |  |  |  |  |  |  |
| *Middle tertile* | 61.584* | 51.445* | 3.790** | 8.174** | 1.109* | 5.586*** | 17.767*** |
| *Highest tertile* | 166.609* | 1 510.108* | 10.609** | 20.159** | 2.125* | 14.287*** | 43.788*** |
| **Human Development Index** |  |  |  |  |  |  |  |
| *Middle tertile, score* | 0.772 | 0.674 | 0.802 | 0.709 | 0.718 | 0.470 | 0.759 |
| *Highest tertile, score* | 0.789 | 0.740 | 0.846 | 0.742 | 0.747 | 0.562 | 0.776 |

**Note.** Gross domestic product is presented in the currency of each country. * Billion; ** Thousands of million. ***Million.

**Supplementary table B**. Meta-analysis of the association between different demographic, macroeconomic, and human development factors and occupational physical activity (third tertile vs. first tertile).

|  | Men | | |  | Women | | |
| --- | --- | --- | --- | --- | --- | --- | --- |
|  | OR (95%CI) | % Weight | *I*^2^ |  | OR (95%CI) | % Weight | *I*^2^ |
| Total population |  |  |  |  |  |  |  |
| Overall | 1.09 (0.92-1.30) | 100.00 | 0.0% (p=0.509) |  | 1.28 (1.11-1.49) | 100.00 | 0.0% (p=0.380) |
| Brazil | 1.13 (0.92-1.38) | 73.45 |  |  | 1.26 (1.07-1.48) | 83.39 |  |
| Chile | 1.14 (0.74-1.76) | 16.74 |  |  | 1.70 (1.07-2.70) | 10.15 |  |
| Peru | 0.80 (0.45-1.40) | 9.80 |  |  | 1.05 (0.59-1.87) | 6.46 |  |
|  |  |  |  |  |  |  |  |
| Population density |  |  |  |  |  |  |  |
| Overall | 0.91 (0.57-1.45) | 100.00 | 81.0% (p=0.005) |  | 1.29 (0.88-1.87) | 100.00 | 69.0% (p=0.040) |
| Brazil | 0.97 (0.80-1.17) | 39.32 |  |  | 1.21 (1.02-1.44) | 45.17 |  |
| Chile | 1.43 (0.96-2.13) | 32.30 |  |  | 1.89 (1.29-2.77) | 32.73 |  |
| Peru | 0.49 (0.30-0.82) | 28.37 |  |  | 0.82 (0.45-1.49) | 22.10 |  |
|  |  |  |  |  |  |  |  |
| Gross Domestic Product |  |  |  |  |  |  |  |
| Overall | 0.89 (0.50-1.59) | 100.00 | 87.4% (p<0.001) |  | 1.24 (0.74-2.08) | 100.00 | 86.7% (p=0.001) |
| Brazil | 1.20 (0.98-1.46) | 37.01 |  |  | 1.48 (1.30-1.68) | 38.86 |  |
| Chile | 1.25 (0.81-1.91) | 32.04 |  |  | 1.98 (1.38-2.84) | 33.28 |  |
| Peru | 0.44 (0.27-0.69) | 30.96 |  |  | 0.56 (0.33-0.96) | 27.86 |  |
|  |  |  |  |  |  |  |  |
| Human Development Pruduct |  |  |  |  |  |  |  |
| Overall | 0.80 (0.45-1.44) | 100.00 | 87.6% (p<0.001) |  | 0.99 (0.64-1.55) | 100.00 | 73.7% (p=0.022) |
| Brazil | 1.12 (0.91-1.39) | 36.69 |  |  | 1.23 (1.03-1.46) | 43.04 |  |
| Chile | 1.03 (0.66-1.61) | 31.46 |  |  | 1.25 (0.75-2.10) | 28.51 |  |
| Peru | 0.42 (0.27-0.65) | 31.85 |  |  | 0.57 (0.34-1.55) | 28.45 |  |

Note. Weights are from random effects analysis. OR, odds ratio. CI, confidence interval. The third tertile represents the highest tertile and the first tertile represents the lowest.

**Supplementary table C**. Meta-analysis of the association between different sociodemographic factors and transport physical activity (second tertile vs. first tertile).

|  | Men | | |  | Women | | |
| --- | --- | --- | --- | --- | --- | --- | --- |
|  | OR (95%CI) | % Weight | I^2^ |  | OR (95%CI) | % Weight | I^2^ |
| Total population |  |  |  |  |  |  |  |
| Overall | 1.31 (1.14-1.51) | 100.00 | 0.0% (p=0.553) |  | 1.36 (1.19-1.55) | 100.00 | 0.0% (p=0.569) |
| Argentina | 1.56 (1.09-2.23) | 14.96 |  |  | 1.30 (0.80-2.10) | 7.94 |  |
| Brazil | 1.22 (0.97-1.55) | 34.46 |  |  | 1.31 (1.07-1.62) | 42.45 |  |
| Chile | 1.27 (0.94-1.71) | 21.58 |  |  | 1.43 (1.06-1.92) | 20.79 |  |
| Colombia | 1.52 (1.12-2.06) | 20.86 |  |  | 1.56 (1.17-2.08) | 22.25 |  |
| Ecuador | 0.90 (0.49-1.64) | 5.26 |  |  | 0.74 (0.36-1.54) | 3.43 |  |
| Peru | 1.06 (0.47-2.40) | 2.88 |  |  | 1.23 (0.57-2.65) | 3.14 |  |
|  |  |  |  |  |  |  |  |
| Population Density |  |  |  |  |  |  |  |
| Overall | 1.28 (1.11-1.47) | 100.00 | 0.0% (p=0.885) |  | 1.25 (1.04-1.51) | 100.00 | 35.9% (p=0.167) |
| Argentina | 1.25 (0.86-1.82) | 14.16 |  |  | 1.13 (0.70-1.81) | 11.61 |  |
| Brazil | 1.31 (1.03-1.68) | 32.35 |  |  | 1.54 (1.26-1.87) | 31.98 |  |
| Chile | 1.37 (1.03-1.83) | 24.25 |  |  | 1.16 (1.88-1.53) | 23.23 |  |
| Colombia | 1.22 (0.89-1.68) | 20.08 |  |  | 1.38 (1.02-1.86) | 21.59 |  |
| Ecuador | 1.25 (0.70-2.22) | 5.94 |  |  | 0.73 (0.36-1.46) | 6.07 |  |
| Peru | 0.80 (0.37-1.76) | 3.22 |  |  | 0.85 (0.41-1.77) | 5.52 |  |
|  |  |  |  |  |  |  |  |
| Gross Domestic Product |  |  |  |  |  |  |  |
| Overall | 1.18 (1.02-1.37) | 100.00 | 0.0% (p=0.773) |  | 1.19 (1.03-1.38) | 100.00 | 0.0% (p=0.801) |
| Argentina | 0.98 (0.65-1.48) | 13.36 |  |  | 1.07 (0.65-1.76) | 8.50 |  |
| Brazil | 1.16 (0.88-1.54) | 29.14 |  |  | 1.19 (0.92-1.52) | 33.80 |  |
| Colombia | 1.40 (1.04-1.89) | 25.33 |  |  | 1.27 (0.95-1.69) | 25.77 |  |
| Ecuador | 1.19 (0.87-1.63) | 22.48 |  |  | 1.22 (0.91-1.64) | 24.24 |  |
| Chile | 0.97 (0.53-1.76) | 6.24 |  |  | 0.74 (0.35-1.54) | 3.90 |  |
| Peru | 1.09 (0.49-2.44) | 3.45 |  |  | 1.46 (0.69-3.10) | 3.79 |  |
|  |  |  |  |  |  |  |  |
| Human Development Pruduct |  |  |  |  |  |  |  |
| Overall | 0.89 (0.73-1.07) | 100.00 | 35.8% (p=0.168) |  | 0.88 (0.74-1.06) | 100.00 | 24.0% (p=0.254) |
| Argentina | 0.99 (0.68-1.44) | 17.09 |  |  | 1.15 (0.72-1.84) | 11.86 |  |
| Brazil | 0.70 (0.55-0.89) | 27.56 |  |  | 0.78 (0.61-0.99) | 30.63 |  |
| Chile | 1.00 (0.71-1.41) | 19.07 |  |  | 0.94 (0.66-0.99) | 18.48 |  |
| Colombia | 1.10 (0.80-1.52) | 20.62 |  |  | 1.07 (0.80-1.43) | 24.68 |  |
| Ecuador | 0.96 (0.53-1.73) | 8.59 |  |  | 0.66 (0.35-1.26) | 6.96 |  |
| Peru | 0.60 (0.31-1.17) | 7.07 |  |  | 0.58 (0.31-1.08) | 7.39 |  |

Note. Weights are from random effects analysis. OR, odds ratio. CI, confidence interval. The second tertile represents the intermediate tertile and the first tertile represents the lowest.

**Supplementary table D**. Meta-analysis of the association between different sociodemographic factors and leisure physical activity (second tertile vs. first tertile).

|  | Men | | |  | Women | | |
| --- | --- | --- | --- | --- | --- | --- | --- |
|  | OR (95%CI) | % Weight | I^2^ |  | OR (95%CI) | % Weight | I^2^ |
| Total population |  |  |  |  |  |  |  |
| Overall | 1.07 (0.92-1.25) | 100.00 | 34.0% (p=0.181) |  | 1.01 (0.89-1.15) | 100.00 | 0.0% (p=0.440) |
| Argentina | 0.92 (0.69-1.23) | 18.40 |  |  | 1.04 (0.74-1.46) | 14.32 |  |
| Brazil | 1.00 (0.86-1.17) | 33.08 |  |  | 1.08 (0.87-1.34) | 36.23 |  |
| Chile | 1.32 (0.89-1.95) | 11.92 |  |  | 1.04 (0.72-1.50) | 12.30 |  |
| Colombia | 1.47 (1.08-2.00) | 16.80 |  |  | 1.14 (0.83-1.57) | 16.58 |  |
| Ecuador | 0.93 (0.65-1.33) | 13.53 |  |  | 0.73 (0.53-1.01) | 15.94 |  |
| Peru | 0.93 (0.52-1.65) | 6.27 |  |  | 1.05 (0.57-1.92) | 4.62 |  |
|  |  |  |  |  |  |  |  |
| Population Density |  |  |  |  |  |  |  |
| Overall | 1.02 (0.85-1.22) | 100.00 | 55.5% (p=0.047) |  | 1.01 (0.84-1.21) | 100.00 | 51.5% (p=0.067) |
| Argentina | 0.92 (0.70-1.21) | 18.46 |  |  | 0.91 (0.66-1.25) | 16.90 |  |
| Brazil | 0.98 (0.84-1.14) | 25.93 |  |  | 1.27 (1.04-1.54) | 24.52 |  |
| Chile | 1.18 (0.85-1.65) | 15.44 |  |  | 0.94 (0.70-1.27) | 17.88 |  |
| Colombia | 1.52 (1.12-2.06) | 16.79 |  |  | 1.24 (0.90-1.71) | 16.67 |  |
| Ecuador | 0.82 (0.59-1.15) | 15.18 |  |  | 0.85 (0.61-1.19) | 16.24 |  |
| Peru | 0.71 (0.41-1.23) | 8.21 |  |  | 0.64 (0.36-1.14) | 7.80 |  |
|  |  |  |  |  |  |  |  |
| Gross Domestic Product |  |  |  |  |  |  |  |
| Overall | 1.17 (1.04-1.33) | 100.00 | 15.6% (p=0.313) |  | 1.07 (0.94-1.21) | 100.00 | 0.0% (p=0.670) |
| Argentina | 1.20 (0.91-1.58) | 16.94 |  |  | 1.10 (0.77-1.57) | 12.66 |  |
| Brazil | 1.17 (1.00-1.36) | 40.66 |  |  | 1.15 (0.94-1.41) | 39.87 |  |
| Colombia | 1.53 (1.11-2.10) | 13.10 |  |  | 1.12 (0.79-1.58) | 13.01 |  |
| Ecuador | 1.22 (0.89-1.68) | 13.30 |  |  | 1.05 (0.77-1.44) | 16.06 |  |
| Chile | 0.86 (0.61-1.21) | 11.57 |  |  | 0.82 (0.58-1.14) | 14.31 |  |
| Peru | 1.05 (0.59-1.87) | 4.43 |  |  | 1.06 (0.57-1.97) | 4.10 |  |
|  |  |  |  |  |  |  |  |
| Human Development Pruduct |  |  |  |  |  |  |  |
| Overall | 1.05 (0.95-1.17) | 100.00 | 0.0% (p=0.750) |  | 1.19 (1.06-1.34) | 100.00 | 0.0% (p=0.588) |
| Argentina | 1.11 (0.86-1.44) | 17.57 |  |  | 1.23 (0.90-1.68) | 14.19 |  |
| Brazil | 1.05 (0.90-1.23) | 48.48 |  |  | 1.21 (1.00-1.46) | 38.46 |  |
| Chile | 1.29 (0.92-1.82) | 9.90 |  |  | 1.42 (1.04-1.94) | 14.61 |  |
| Colombia | 0.91 (0.66-1.25) | 11.47 |  |  | 0.93 (0.68-1.27) | 14.35 |  |
| Ecuador | 0.97 (0.68-1.39) | 9.03 |  |  | 1.20 (0.88-1.64) | 14.75 |  |
| Peru | 0.94 (0.53-1.67) | 3.56 |  |  | 1.17 (0.63-2.19) | 3.65 |  |

Note. Weights are from random effects analysis. OR, odds ratio. CI, confidence interval. The second tertile represents the intermediate tertile and the first tertile represents the lowest.

**Supplementary table E**. Meta-analysis of the association between different sociodemographic factors and habitual physical activity (second tertile vs. first tertile).

|  | Men | | |  | Women | | |
| --- | --- | --- | --- | --- | --- | --- | --- |
|  | OR (95%CI) | % Weight | I^2^ |  | OR (95%CI) | % Weight | I^2^ |
| Total population |  |  |  |  |  |  |  |
| Overall | 1.20 (1.02-1.42) | 100.00 | 41.4% (p=0.115) |  | 1.16 (1.03-1.31) | 100.00 | 0.0% (p=0.824) |
| Argentina | 1.07 (0.75-1.52) | 13.62 |  |  | 1.05 (0.71-1.54) | 9.62 |  |
| Brazil | 1.07 (0.90-1.26) | 27.29 |  |  | 1.13 (0.96-1.34) | 52.49 |  |
| Chile | 1.39 (1.05-1.86) | 17.67 |  |  | 1.40 (0.97-2.03) | 10.27 |  |
| Colombia | 1.56 (1.21-2.01) | 20.05 |  |  | 1.30 (0.94-1.79) | 13.33 |  |
| Ecuador | 1.26 (0.84-1.89) | 11.41 |  |  | 1.12 (0.71-1.76) | 6.75 |  |
| Peru | 1.08 (0.48-2.43) | 3.65 |  |  | 1.37 (0.69-2.74) | 2.94 |  |
| Uruguay | 0.74 (0.40-1.33) | 6.31 |  |  | 0.89 (0.51-1.55) | 4.61 |  |
|  |  |  |  |  |  |  |  |
| Population Density |  |  |  |  |  |  |  |
| Overall | 1.12 (0.96-1.32) | 100.00 | 39.1% (p=0.131) |  | 1.05 (0.85-1.30) | 100.00 | 63.3% (p=0.012) |
| Argentina | 0.98 (0.69-1.40) | 13.72 |  |  | 0.81 (0.57-1.16) | 14.76 |  |
| Brazil | 1.20 (1.02-1.41) | 28.20 |  |  | 1.42 (1.23-1.64) | 22.46 |  |
| Chile | 1.16 (0.89-1.52) | 18.59 |  |  | 0.93 (0.68-1.29) | 15.89 |  |
| Colombia | 1.47 (1.12-1.92) | 18.71 |  |  | 1.34 (0.97-1.85) | 15.84 |  |
| Ecuador | 0.97 (0.64-1.45) | 11.21 |  |  | 0.99 (0.62-1.58) | 11.31 |  |
| Peru | 0.52 (0.25-1.06) | 4.41 |  |  | 0.82 (0.43-1.56) | 7.63 |  |
| Uruguay | 1.02 (0.53-1.96) | 5.16 |  |  | 0.89 (0.57-1.38) | 12.10 |  |
|  |  |  |  |  |  |  |  |
| Gross Domestic Product |  |  |  |  |  |  |  |
| Overall | 1.16 (1.02-1.32) | 100.00 | 14.8% (p=0.317) |  | 1.11 (0.99-1.24) | 100.00 | 1.2% (p=0.415) |
| Argentina | 0.94 (0.66-1.35) | 11.01 |  |  | 1.01 (0.69-1.48) | 8.37 |  |
| Brazil | 1.19 (1.02-1.40) | 37.44 |  |  | 1.18 (1.01-1.38) | 49.41 |  |
| Colombia | 1.30 (0.99-1.72) | 16.73 |  |  | 1.15 (0.87-1.53) | 15.23 |  |
| Ecuador | 1.40 (1.07-1.82) | 18.29 |  |  | 1.26 (0.91-1.73) | 11.95 |  |
| Chile | 0.93 (0.63-1.37) | 9.38 |  |  | 0.79 (0.52-1.21) | 6.86 |  |
| Peru | 0.74 (0.35-1.57) | 2.71 |  |  | 1.04 (0.53-2.04) | 2.73 |  |
| Uruguay | 0.95 (0.53-1.70) | 4.45 |  |  | 0.78 (0.49-1.26) | 5.44 |  |
|  |  |  |  |  |  |  |  |
| Human Development Pruduct |  |  |  |  |  |  |  |
| Overall | 0.97 (0.86-1.11) | 100.00 | 15.2% (p=0.314) |  | 1.00 (0.87-1.16) | 100.00 | 15.8% (p=0.309) |
| Argentina | 1.13 (0.81-1.56) | 13.31 |  |  | 1.25 (0.88-1.78) | 14.13 |  |
| Brazil | 0.92 (0.78-1.10) | 34.87 |  |  | 0.94 (0.79-1.13) | 37.08 |  |
| Chile | 0.94 (0.71-1.25) | 16.52 |  |  | 1.15 (0.76-1.74) | 10.69 |  |
| Colombia | 0.98 (0.74-1.31) | 16.46 |  |  | 1.14 (0.83-1.58) | 16.38 |  |
| Ecuador | 0.85 (0.56-1.29) | 8.70 |  |  | 1.04 (0.64-1.69) | 8.14 |  |
| Peru | 0.65 (0.35-1.22) | 4.11 |  |  | 0.57 (0.32-1.03) | 5.67 |  |
| Uruguay | 1.63 (0.98-2.70) | 6.01 |  |  | 0.83 (0.51-1.35) | 7.91 |  |

Note. Weights are from random effects analysis. OR, odds ratio. CI, confidence interval. The second tertile represents the intermediate tertile and the first tertile represents the lowest.

**Supplementary table F**. Meta-analysis of the association between different sociodemographic factors and occupational physical activity (second tertile vs. first tertile)

|  | Men | | |  | Women | | |
| --- | --- | --- | --- | --- | --- | --- | --- |
|  | OR (95%CI) | % Weight | I^2^ |  | OR (95%CI) | % Weight | I^2^ |
| Total population |  |  |  |  |  |  |  |
| Overall | 1.03 (0.86-1.23) | 100.00 | 0.0% (p=0.634) |  | 0.99 (0.85-1.15) | 100.00 | 0.0% (p=0.459) |
| Brazil | 1.09 (0.88-1.34) | 72.50 |  |  | 0.97 (0.82-1.14) | 81.63 |  |
| Chile | 0.88 (0.57-1.34) | 17.05 |  |  | 1.30 (0.82-2.05) | 11.03 |  |
| Peru | 0.94 (0.54-1.62) | 10.45 |  |  | 0.89 (0.51-1.15) | 7.34 |  |
|  |  |  |  |  |  |  |  |
| Population density |  |  |  |  |  |  |  |
| Overall | 0.88 (0.59-1.32) | 100.00 | 77.8% (p=0.011) |  | 1.11 (0.85-1.45) | 100.00 | 50.7% (p=0.132) |
| Brazil | 1.19 (0.99-1.44) | 39.93 |  |  | 1.30 (1.10-1.55) | 51.51 |  |
| Chile | 0.86 (0.60-1.22) | 32.60 |  |  | 0.89 (0.63-1.26) | 30.85 |  |
| Peru | 0.58 (0.36-0.93) | 27.47 |  |  | 1.01 (0.59-1.75) | 17.65 |  |
|  |  |  |  |  |  |  |  |
| Gross Domestic Product |  |  |  |  |  |  |  |
| Overall | 0.91 (0.66-1.25) | 100.00 | 60.4% (p=0.080) |  | 0.99 (0.77-1.28) | 100.00 | 49.6% (p=0.138) |
| Brazil | 1.11 (0.91-1.35) | 45.45 |  |  | 1.11 (0.97-1.28) | 54.61 |  |
| Chile | 0.92 (0.60-1.39) | 28.59 |  |  | 1.02 (0.71-1.47) | 27.84 |  |
| Peru | 0.63 (0.40-1.00) | 25.96 |  |  | 0.65 (0.39-1.09) | 17.56 |  |
|  |  |  |  |  |  |  |  |
| Human Development Pruduct |  |  |  |  |  |  |  |
| Overall | 0.90 (0.65-1.24) | 100.00 | 61.8% (p=0.073) |  | 0.92 (0.71-1.20) | 100.00 | 35.8% (p=0.211) |
| Brazil | 1.05 (0.86-1.28) | 44.95 |  |  | 0.94 (0.79-1.11) | 60.72 |  |
| Chile | 1.03 (0.66-1.60) | 27.24 |  |  | 1.25 (0.74-2.09) | 19.66 |  |
| Peru | 0.60 (0.39-0.93) | 27.81 |  |  | 0.65 (0.39-1.09) | 19.62 |  |

Note. Weights are from random effects analysis. OR, odds ratio. CI, confidence interval. The second tertile represents the intermediate tertile and the first tertile represents the lowest.

**Supplementary table G**. Meta-analysis of the association between different sociodemographic factors and sitting time (second tertile vs. first tertile).

|  | Men | | |  | Women | | |
| --- | --- | --- | --- | --- | --- | --- | --- |
|  | OR (95%CI) | % Weight | I^2^ |  | OR (95%CI) | % Weight | I^2^ |
| Total population |  |  |  |  |  |  |  |
| Overall | 1.00 (0.74-1.36) | 100.00 | 62.8% (p=0.045) |  | 1.00 (0.72-1.38) | 100.00 | 66.3% (p=0.031) |
| Argentina | 0.78 (0.64-0.95) | 38.98 |  |  | 0.74 (0.58-0.94) | 34.66 |  |
| Brazil | 0.98 (0.75-1.27) | 34.85 |  |  | 0.97 (0.75-1.25) | 34.02 |  |
| Chile | 1.30 (0.81-2.08) | 22.13 |  |  | 1.26 (0.87-1.84) | 27.18 |  |
| Peru | 3.48 (0.81-14.91) | 4.04 |  |  | 3.45 (0.76-15.55) | 4.13 |  |
|  |  |  |  |  |  |  |  |
| Population Density |  |  |  |  |  |  |  |
| Overall | 0.85 (0.72-1.00) | 100.00 | 13.9% (p=0.323) |  | 0.88 (0.75-1.03) | 100.00 | 3.7% (p=0.374) |
| Argentina | 0.78 (0.65-0.94) | 52.19 |  |  | 0.78 (0.62-0.98) | 43.86 |  |
| Brazil | 0.93 (0.73-1.18) | 35.42 |  |  | 0.93 (0.73-1.19) | 38.72 |  |
| Chile | 1.05 (0.66-1.68) | 11.17 |  |  | 1.10 (0.75-1.61) | 16.36 |  |
| Peru | 0.35 (0.08-1.51) | 1.23 |  |  | 0.52 (0.11-2.36) | 1.06 |  |
|  |  |  |  |  |  |  |  |
| Gross Domestic Product |  |  |  |  |  |  |  |
| Overall | 1.06 (0.78-1.46) | 100.00 | 66.1% (p=0.031) |  | 1.07 (0.87-1.33) | 100.00 | 28.9% (p=0.239) |
| Argentina | 1.35 (1.12-1.63) | 38.99 |  |  | 1.24 (0.95-1.60) | 37.78 |  |
| Brazil | 0.87 (0.67-1.13) | 34.78 |  |  | 0.90 (0.70-1.16) | 39.06 |  |
| Chile | 0.87 (0.54-1.41) | 22.13 |  |  | 1.06 (0.71-1.59) | 21.16 |  |
| Peru | 1.73 (0.39-7.66) | 4.10 |  |  | 2.52 (0.57-11.20) | 2.01 |  |
|  |  |  |  |  |  |  |  |
| Human Development Pruduct |  |  |  |  |  |  |  |
| Overall | 1.01 (0.87-1.18) | 100.00 | 0.0% (p=0.516) |  | 1.03 (0.88-1.21) | 100.00 | 0.0% (p=0.566) |
| Argentina | 1.12 (0.92-1.38) |  |  |  | 1.15 (0.89-1.48) | 39.21 |  |
| Brazil | 0.92 (0.72-1.18) |  |  |  | 0.90 (0.71-1.15) | 42.50 |  |
| Chile | 0.83 (0.51-1.33) |  |  |  | 1.10 (0.75-1.61) | 17.28 |  |
| Peru | 1.05 (0.22-4.94) |  |  |  | 1.30 (0.27-6.34) | 1.01 |  |

Note. Weights are from random effects analysis. OR, odds ratio. CI, confidence interval. The second tertile represents the intermediate tertile and the first tertile represents the lowest.
